# A Two-Stage Decision Framework for Resolving Brownfield Conflicts

**DOI:** 10.3390/ijerph16061039

**Published:** 2019-03-22

**Authors:** Qingye Han, Yuming Zhu, Ginger Y. Ke, Hongli Lin

**Affiliations:** 1 School of Management, Northwestern Polytechnical University, Xi’an 710100, China; hanqy11@mail.nwpu.edu.cn (Q.H.); zym1886@nwpu.edu.cn (Y.Z.); lhl2016@mail.nwpu.edu.cn (H.L.); 2 Faculty of Business Administration, Memorial University of Newfoundland, St. John’s, NL A1B 3X9, Canada

**Keywords:** graph model of conflict resolution, brownfields, inverse problem, negotiation, third party intervention

## Abstract

Based on the Graph Model of Conflict Resolution (GMCR), a two-stage decision framework is developed to reveal the essence of brownfield incidents and facilitate the resolution of brownfield conflicts caused by the incidents. More particularly, the forward GMCR is utilized in Stage I, the negotiation stage, to simulate the evolution of a Brownfield Conflict (BC) and predict its potential resolution via stability analysis. If no acceptable equilibrium can be obtained, the BC progresses into Stage II, the third-party-intervention stage, where the inverse GMCR is used to assist a third party in intervening the conflict to achieve a desirable outcome. To illustrate the practicality of this framework, a recent BC that occurred in Changzhou, China, is taken as a case study. Invaluable insights are provided through the computation and investigation of the corresponding preference relationships.

## 1. Introduction

A brownfield generally refers to a property, whose reuse, redevelopment, or expansion may be complicated due to the existence or potential existence of pollutants or other hazardous substances [1]. Global industrialization has led to a large quantity of brownfields, which have been posing high risk to both human health and the environment. It is estimated that the amount of brownfields in the United States is between 0.5 and one million [2]. From 2001–2015 in China, over 100,000 factories were located elsewhere, which in turn led to about two million hectares of brownfield in the major cities [3]. What makes it even worse is that more brownfields are being created along with the industrial upgrading process [4]. The reuse of brownfields is a sustainable land use strategy and brings about a wide range of benefits for the economy, environment, and society [5,6]. In most industrialized countries, the recreation of brownfields is in a very high priority position on their political agendas [7,8]. A series of measures, such as relevant legal systems, remediation standards, and economic incentives, have been taken into practice.

However, the reuse progress of brownfields is still slow and problematic. A large number of brownfield-related crises with respect to the environment, ecology, human health, or even society have been reported. The “Love Canal” disaster, one of the most shocking environmental tragedies in the United States, attracted broad attention for the public health problem caused by the massive dumping of toxic waste in the ground [9]. A dispute in Elmira, Ontario, Canada, was induced by the pollution of the aquifer by a chemical plant [10]. The collective poisoning incident of students in Changzhou, which arose from an explosion of second-hand pollution during the remediation of brownfields, was reported by the most famous channel (China Central Television, CCTV) in China [11]. These brownfield-related crises have led to numerous and intense conflicts among multiple stakeholders. Generally, these type of incidents are sensitive and complicated, easily escalating to severe disputes. A decision framework therefore is urgently needed to support resolving brownfield conflicts.

Three types of analytical tools have been utilized to model, predict, and resolve conflicts formally. The first cluster is the decision making theory. For instance, Maguire and Boiney [12] analyzed an environmental dispute based on the decision making tree, and Driscoll et al. [13] used the multiple criteria decision making method to resolve management conflicts of future fire. The second group integrated the cost-benefit analysis to support conflict resolution. For example, Gebken and Gibson [14] compared existing dispute resolution methods depending on their transaction cost. Supported by utility theory, Cheung and Suen [15] selected the best dispute resolution strategy, and Zeleznikow et al. [16] built a utility function to help with disputes’ resolution through negotiation. The third and most widely-used method is the game theory introduced by Von Neumann and Morgenstern [17]. Recent examples include Blokhuis et al. [7], who utilized the classical game theory and conjoint analysis together to predict the government’s and redeveloper’s choice between conflict and cooperation within the brownfield redevelopment projects; and Glumac et al. [18,19], who designed a model to investigate the negotiation process between a developer and a municipality when building the public-private partnership in brownfield redevelopment projects. Under the regime of game theory, the Graph Model of Conflict Resolution (GMCR), first introduced by Fraser and Hipel [20] and then improved and comprehended by Kilgour [21], Thomas [22], Fang et al. [23], and Fang et al. [24], was purposefully proposed to resolve real-world disputes formally. GMCR has several advantages over the classical game theory techniques: requiring much less input information from the user and allowing decision makers to move in any possible order, which is more realistic in conflict analysis. Due to the benefit of GMCR, our systematic Brownfield Conflict (BC) resolution framework is developed on the basis of this methodology.

Researchers have tried to model BC incidents with GMCR. Wang et al. [25] used GMCR to model and analyze the conflict in brownfield redevelopment for forming the strategic perspective of policy making. Hipel et al. [2] developed a negotiation methodology for resolving the brownfield redevelopment issues by providing strategic and tactical management insights. Bashar et al. [26] integrated fuzzy preferences in the GMCR to identify possible solutions of brownfields’ redevelopment conflicts considering the situation of uncertain preference information. Walker et al. [27] defined a matrix representation of GMCR, which was applied to negotiate the acquisition of a brownfield property from a property owner to a developer. Kuang et al. [28] proposed a grey-based GMCR model to simulate human behaviors in a brownfield redevelopment conflict featuring uncertainty. The work by Wang et al. [29] was comprised of the ordered weighted averaging, fuzzy real options, and GMCR in a framework to facilitate risky project negotiation, and a brownfield redevelopment case was used to test the framework. Han et al. [30] examined the financing dilemma of brownfield remediation problems in China with GMCR. Philpot et al. [31] detected new strategic insights for the dispute in Elmira, Ontario, using GMCR considering the recent situation in 2016. Yin et al. [32] produced an improved GMCR by integrated a score function, and this improved GMCR was applied in the “Changzhou Foreign Language School Incident”. Yu and Pei [8] analyzed a case in China by using GMCR with the preference strength taken into account.

Four aspects distinguish this paper from other studies. First, most researchers focused on conflicts in the redevelopment phase of brownfields, while this paper analyzes possible disputes during the whole lifecycle of brownfields. Second, the above studies generally only discuss an economy or cooperation issue, whereas this research also addresses the environment and health conflicts induced by brownfields. Third, most research introduced a negotiation model to solve a brownfield dispute without considering the situation that the stakeholders may not be able to resolve it by themselves. However, this study designs a two-stage framework where Stage II introduces how a third party can intervene in a brownfield conflict and obtain the implications from inverse GMCR analysis. Last, but not least, all existing research only used BCs as examples to verify certain improvements of GMCR. The present paper, however, investigates BCs from the viewpoint of the nature of the conflicts and correspondingly proposes a decision framework particularly designed for resolving this type of dispute. In more detail, the objectives of this paper are as follows:

(1) designing a framework to support the resolutions of BCs systematically, so as to avoid unnecessary conflict escalations;
(2) modeling BC to predict the possible outcomes and show the corresponding evolution paths;
(3) introducing a third-party-intervention mechanism when the stakeholders of BCs cannot reach acceptable resolutions by themselves;
(4) implementing the framework for a collective poisoning incident in China to show the effectiveness.

The rest of this paper is organized as follows. The fundamental theory of GMCR is introduced in Section 2, followed by the presented framework in Section 3. A real brownfield conflict that occurred in China is summarized in Section 4 to demonstrate the effectiveness of the framework. Section 4.3 conducts a discussion on how to provide practical implications based on the required preference. Section 5 concludes the present paper and addresses the future research directions.

## 2. Methodologies

### 2.1. Graph Model For Conflict Resolution

The structure of GMCR consists of four main components [23,33].

- A set of stakeholders or decision makers, denoted as $N=\left\{1,2\ldots i\ldots n\right\}$.
- Sets of all decision makers’ available options, denoted as $O_{i}=\left\{o_{i1},o_{i2}\ldots \right\}$.
- A set of feasible states, denoted as $S=\left\{s_{1},s_{2}\ldots s_{m}\right\}$.
- A set of preferences among the above-mentioned states, denoted as $P=\left\{P_{i}\right\}$, where $P_{i}$ represents the preferences of decision maker *i*. A couple of binary relations, $\left\{{≻}_{i},{∼}_{i}\right\}$, are utilized to express the preferences. For example, $s_{1}{≻}_{i}s_{2}$ and $s_{1}{∼}_{i}s_{2}$ respectively indicate that $s_{1}$ is preferred more than $s_{2}$, and $s_{1}$ is the indifference toward $s_{2}$ for decision maker *i*. It should be noted that the relation of ${≻}_{i}$ is asymmetric, while ${∼}_{i}$ is symmetric. Because of the transitive characteristic of the two relations, all states can be ordered from the perspective of each decision maker’s preferences.
- A directed graph consisting of available moves, denoted as $\left\{M_{i}=\left(S,A_{i}\right)\right\}$, where $A_{i}$ is the set of directed arcs of decision maker *i*. Each arc in the graph represents a move from one state to another adjacent state.

Stability concepts mathematically define the possible stable resolutions of a conflict. GMCR commonly employs four stability concepts, including Nash Stability (Nash) [34], General Metarationality (GMR) [35], Symmetric Metarationality (SMR) [35], and Sequential Stability (SEQ) [36]. The corresponding definitions are given as follows.

- Nash: A state is Nash stable if a decision maker cannot leave it for a preferred one unilaterally.
- GMR: A state is GMR stable if all of a decision maker’s unilateral improvements can be sanctioned by other decision makers’ subsequent unilateral moves.
- SMR: A state is SMR stable if all of a decision maker’s unilateral improvements can still be sanctioned by other decision makers, after a potential response from the main decision maker.
- SEQ: A state is SEQ stable if all of a decision maker’s unilateral improvements can be sanctioned by other decision makers.

**Figure 1:**
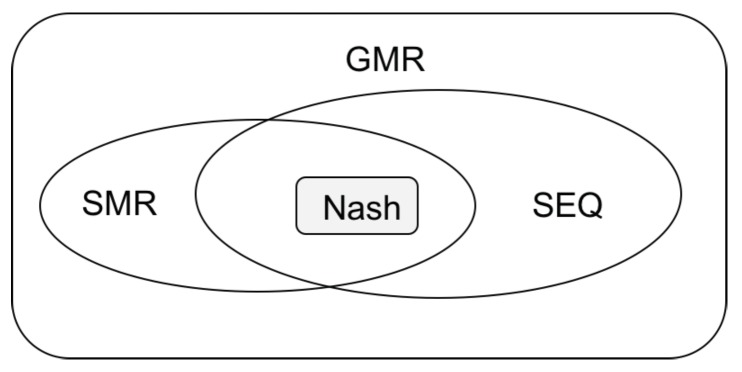
The relationships of the four stabilities.

The characteristics of these stabilities are summarized in Table 1. The “Preference Information” refers to how much information is needed to identify the corresponding stability. For Nash, decision makers only need their own preference information, while all preference information is needed for each decision maker in other three stabilities. The “Foresight” represents the max-count of foreseen moves of a decision maker. Nash checks one move in advance; both GMR and SEQ check two moves in advance; and SMR checks three moves in advance. The “Disimprovement” indicates a decision maker’s tendency to move to a relatively less preferred state for the purpose of reaching a relatively more preferred state. No disimprovement is allowed in Nash and SEQ, while sanctions from other decision makers can be disimprovements in GMR and SMR. The relationships of the above-mentioned four stabilities are shown in Figure 1. It should be noted that Nash is the most stable equilibrium because if a state is Nash, all other stabilities hold.

### 2.2. Inverse GMCR

**Figure 2:**
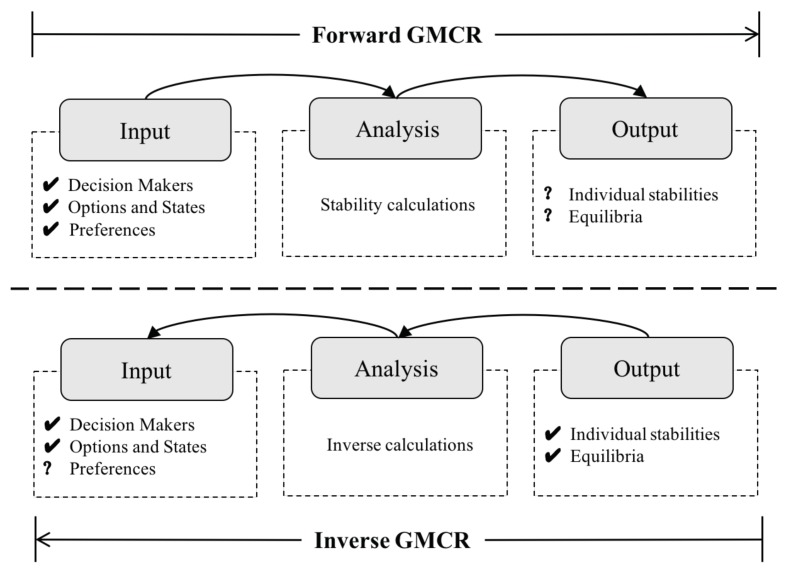
The logic comparison of forward and inverse Graph Model of Conflict Resolution (GMCR) (adapted from [41,42]).

The inverse GMCR [39,40,41] is expended from the above classic GMCR. When the purpose of a user is to model a dispute and predict possible equilibria by the analysis engine, a forward analysis process can be applied. Hence, the classic GMCR is renamed as the forward GMCR here. On the other hand, an inverse GMCR analysis process is more appropriate when the user’s perspective is to determine the required preferences to reach a certain desired equilibrium for decision makers. The aforementioned comparison of perspectives is illustrated in Figure 2, where a check sign (√) depicts the known information and a question mark (?) means the corresponding item needs to be determined.

A more detailed comparison between the forward and inverse GMCR is summarized in Table 2, in terms of purpose, required preferences, analysis type, output, and sensitivity analysis. Compared to the aim of forward GMCR for predicting the possible equilibria, the inverse GMCR supposes desired equilibria and examines how to reach them from the viewpoint of inverse engineering. A critical feature of inverse GMCR is that it does not require preference information to model the conflict; rather, the preferences are derived from the analysis of desired outcomes. The forward GMCR supports decision making by furnishing possible equilibria, while the inverse GMCR provides required preferences. Furthermore, independent sensitivity analysis, such as status quo analysis and evolution path analysis, is necessary for the forward GMCR; whereas it is integrated by nature for the inverse GMCR.

### 2.3. Software to Support the Application of This Method

Manually solving a problem by using GMCR is possible, yet it may be time consuming, error-prone, and tiresome [43]. Several Decision Support Systems (DSSs) have been developed to facilitate the utilization of GMCR. The first one is “GMCR I” (1990), which is rarely used because of its unfriendly interface, quite limited maximum states, and plain text results. To overcome these drawbacks, “GMCR II” [44] was designed and is known to have a friendly graphical user interface and a powerful analysis engine. The missing inverse concept in “GMCR II” led to the development of “GMCR +” [45], which not only makes the DSS easier to use, but also added new extensions, especially the inverse GMCR function. A comparison chart of “GMCR II” and “GMCR +” can be found in Kinsara [43]. The present paper employs the latest “GMCR +” to assist in resolving BCs through the forward and inverse GMCR processes.

## 3. A Decision Framework for BC Resolution

### 3.1. Brownfield-Related Disputes and Available Resolutions

**Figure 3:**
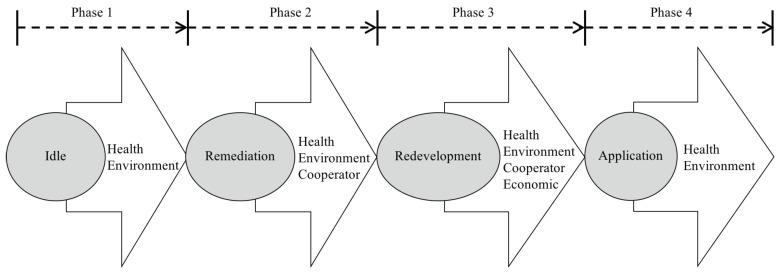
Potential conflicts in different stages of the entire lifecycle.

The lifecycle of a brownfield can be divided into four phases, namely the idle, remediation, redevelopment, and application (Figure 3). Conflicts may occur in any phase. The main motives for the outbreak of brownfield-related conflicts can be summarized as the health, environment, cooperation, and economy reasons. As indicated in Figure 3, the causes for potential conflicts may differ from each other in different phases. BCs are generally complicated, and even worse, multiple conflicts usually take place simultaneously. Note that the cooperative and economic disputes may only occur during the remediation and redevelopment phases because these two phases usually involve multiple stakeholders, such as owners, municipalities, lenders, redevelopers, and purchasers. However, environmental and health conflicts may occur in the entire lifecycle, because brownfields remediation is to match the health or environmental risk with the corresponding functionality.

**Figure 4:**
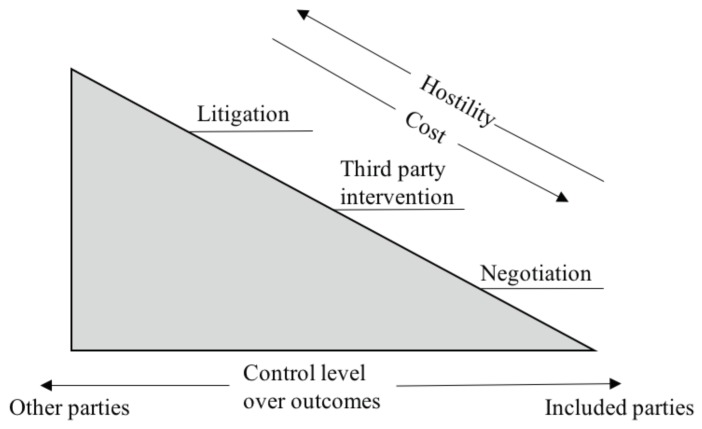
Features of dispute resolution methods (adapted from [2]).

When a brownfield-related dispute occurs, measures and counter measures need to be taken by relevant stakeholders. A negotiation process, because of the low cost and straightforward procedure, is usually taken first to reach a possible agreement. However, an acceptable agreement may not be able to be reached, especially in the environmental and health disputes due to the associated strong conflicts of interest. In this case, other independent parties, namely third parties, may be involved to intervene in the dispute so as to achieve an outcome that is acceptable to all stakeholders. If the BC still cannot be resolved with the third-party intervention, it inevitably enters into a litigation process, which mandatorily resolves the dispute through lawsuits. Features of the three conflict resolution methods can be seen in Figure 4. Negotiation is the lease expensive and most amicable way to resolve a dispute; the third party intervention takes second place; and litigation is the last. As for the control level over outcomes, the stakeholders take charge overall in the negotiation, while the litigation process is totally out of the control of the stakeholders. The third party intervention lays between the other two methods.

### 3.2. A Two-Stage Decision Making Framework

**Figure 5:**
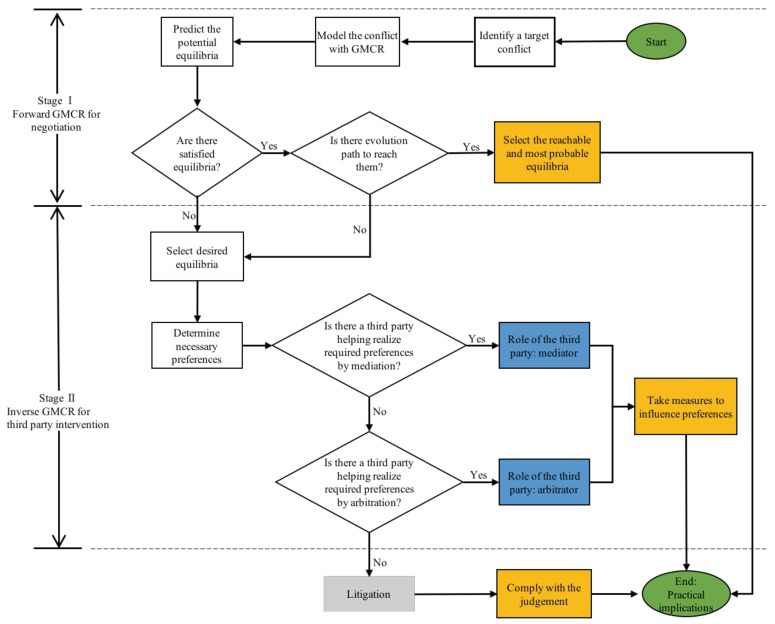
A decision making framework for Brownfield Conflict (BC) resolution.

Herein, a systematic framework (Figure 5) is introduced to support and facilitate the resolution of BC. As shown, the framework contains two stages, which respectively refer to the negotiation and third-party intervention stages. The major purpose of this framework is to use GMCR as an effective tool applied to the two stages, such that the nature of the dispute can be analyzed and understood in order to seek a reasonable outcome at the first stage, and certain desired resolution(s) can be proposed to resolve practically the conflict at the second stage if no agreement can be achieved in the first stage.

More specifically in this framework, the involved parties communicate with each other to resolve differences and so to reach a consensus at the negotiation stage. The forward GMCR in Stage I is used to model the conflict and predict possible equilibria. One or more evolution paths from status quo to the equilibria may also be provided to judge the reachability. The reachable equilibria address information of negotiation results with the highest possibilities, and evolution paths depict how to reach those results. If a reachable and acceptable result exists, the dispute can be resolved by the stakeholders themselves through negotiation. Otherwise, the conflict proceeds into Stage II, where suitable third parties intervene in the BC to assist in achieving a resolution.

In Stage II, the inverse GMCR is used to support the third parties to intervene in the unresolved dispute proceeded from Stage I. One or more desired equilibria, which can be accepted by all stakeholders, are suggested by the third parties. Then, the required and necessary adjustments of preferences for each decision maker can be determined through the inverse GMCR, so as to provide the theoretical basis for the third party intervention. Another critical issue in this stage is to identify a suitable third party that is able to induce the preference adjustments. In a specific industry, there may be corresponding organizations and procedures to resolve disputes. For instance, conflict resolution techniques in the construction industry include partnering, dispute review boards, mediation, arbitration, mini-trail, etc. [46]. Some of these techniques, such as the dispute review board, can only be used in the construction industry, while some others, such as mediation and arbitration, are commonly used in any industry. In this paper, we pay special attention to these two when a third party intervenes in a BC. In more detail, when the third party assists in resolving the dispute through the use of specialized communication and facilitation techniques, the party is called a mediator; while the third party acts as an arbitrator if the resolution of the intervention is legally or politically binding on all stakeholders. In general, a mediation is easier to conduct than an arbitration. Depending on the confrontation degree of BC, the stakeholders determine which approach is more suitable. Potential candidates of the third parties for brownfield-related disputes include environmental non-profit organizations, soil expert teams, government departments, the arbitral institutions of the court, etc. Among the four parties, the first two are relatively more professional, and the last two are relatively more powerful. If no suitable third party can provide a preferable resolution, the dispute most likely proceeds to a lawsuit.

## 4. Case Study

**Figure 6:**
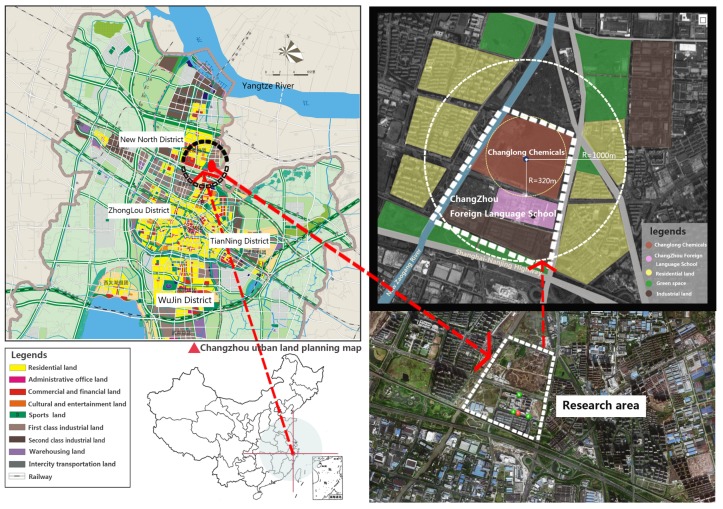
Location of the research target (adapted from [11]).

**Figure 7:**
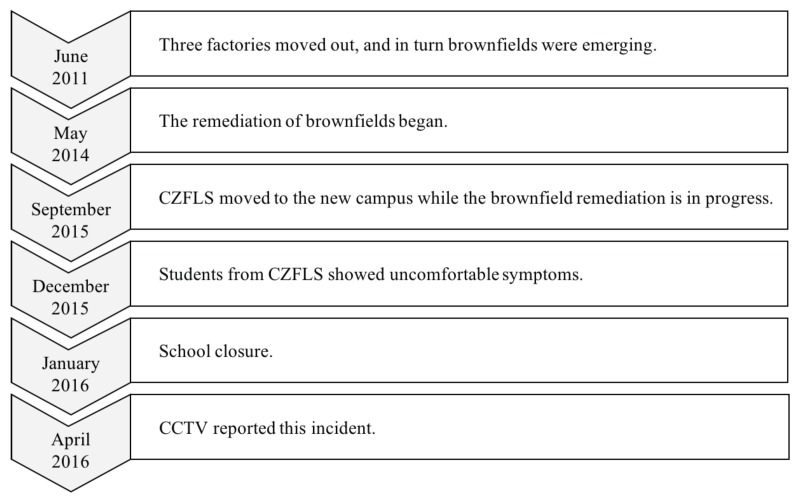
The course of the incident. CCTV, China Central Television.

In this section, a real-world BC that occurred in Changzhou, China, is used to illustrate the implementation of the proposed decision framework. The Changzhou Foreign Language School (CFLS) is a prestigious high school situated in Changzhou, a southeastern city in China (shown in Figure 6). In December 2015, only three months after moving to a new campus, more than 400 students showed special pathological manifestations. Their parents suspected that the situation was related to the secondary pollution from the nearby ongoing brownfield (the Changlong site) remediation project [47]. The unremitting protest was reported by the China Central Television (CCTV), the official TV channel of China. This incident had attracted widespread attention and led the public to realize the severity of urban land pollution problems. Several months following this toxic incident, the first state-level operable plan for soil management in China, the Action Plan for Prevention and Control of Soil Pollution (the Ministry of Environmental Protection of the People’s Republic of China, 2016), was released. The key time points of these incidents are listed in Figure 7.

Despite the fact that the event led to multiple disagreements among different parties, we focus on the health conflict because of the corresponding high level of social attention and the complication in achieving an acceptable resolution. The conflicts of interest made it impossible to resolve the dispute by negotiations among stakeholders. Therefore, the officers from a higher level authority, including Jiangsu Province and the central government, were pointed out as a third party to intervene in this dispute. To show the possibility and effectiveness of the proposed framework, this conflict is modeled in Stage I when it happened. Stage II indicates how the third party induces the dispute to be resolved. In the following subsections, the professional software “GMCR+” was utilized to demonstrate the implementation of the framework.

### 4.1. Stage I: GMCR for Negotiation

Stakeholders of this health conflict consist of students and their parents (DM 1), CFLS (DM 2), Changzhou municipality (DM 3), and the Black Peony Company (BPC), which is responsible for the remediation of the brownfield (DM 4). At this stage, students from CFLS are faced with health risks. As a response, their parents may take a range of measures to protect their children ($O_{1}$). In order to ensure the health of the students, the school can suspend courses in the new campus ($O_{2}$). The Changzhou municipality has two options: relocate the school to another safe site so that the health risk can be completely eliminated ($O_{3}$); or request the BPC to improve the status quo, otherwise punish this company ($O_{4}$). BPC may improve the remediation process under the pressure of public opinion ($O_{5}$). These options are explained in Table 3.

**Figure 8:**
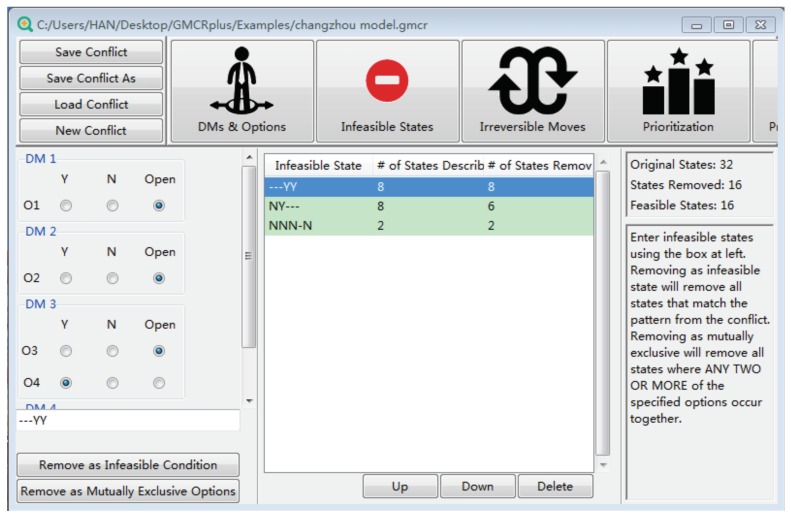
The process of removing infeasible states.

For each option, decision makers may select it or not. As a result, the total combination of options, i.e., the number of states, is 32 ($=2^{5}$) results. Note that certain states may be infeasible and therefore should be eliminated. As indicated in Figure 8, three groups of states need to be deleted: (1) DM 4 is punished when it decides to improve the situation (“−−−YY”); (2) when DM 1 takes no safeguard measures, DM 2 chooses to suspend courses (“NY−−−”); and (3) both DM 1 and DM 2 take no measures when DM 3 does not relocate the campus, and DM 4 does not improve the status quo (“NNN−N”). In the above statement, “*Y*” means the corresponding options being chosen, “*N*” indicates the corresponding options not being chosen, and “−” represents that the option can be either chosen or not. After eliminating infeasible states, “GMCR +” can generate a list including 16 feasible states, as shown in Table 4.

**Figure 9:**
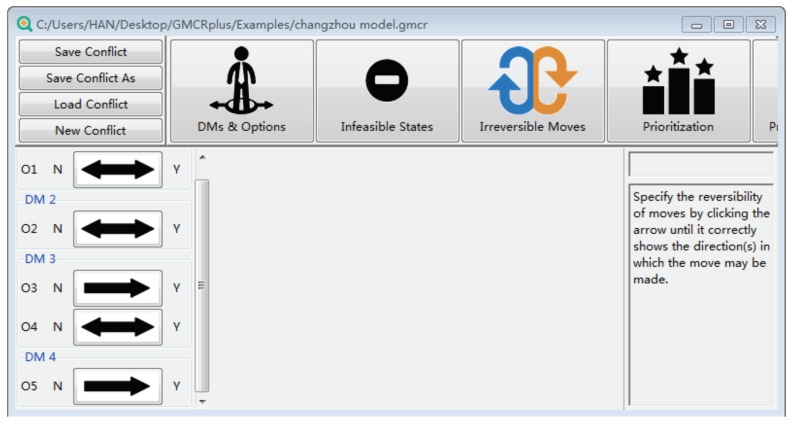
Indication of irreversible moves.

Figure 9 indicates the irreversible moves of this conflict in “GMCR +”, where double-sided arrows represent reversible moves from one option to another, while the one-way arrows show the only direction of moves. For example, DM 3 can only move from not choosing $O_{3}$ to choosing it, but not vice versa.

In “GMCR+”, three techniques, including “option prioritizing”, “option weighting”, and “direct ranking”, can be used to achieve the relative preferences of each decision maker. We herein employ the first one for its simplicity and effectiveness. Table 5 illustrates the order of preference prioritization from the most to least for each decision maker. Take the third column (DM 2) for example. DM 2 most prefers the campus can be relocated, and so, the highest prioritized option is $O_{3}$; next, DM 2 prefers that the status quo can be improved by BPC ($O_{5}$), followed by $O_{4}$, where BPC should be punished; then,“2IF(-3)” means that the courses are suspended ($O_{2}$) if the campus cannot be relocated (not $O_{3}$); and “2IF(-5)” refers to the situation that the courses are suspended ($O_{2}$) if the status cannot be improved by DM 4 (not $O_{5}$). For more a detailed algorithm of this ranking process, please check Fang et al. [24]. Based on the information in Table 5, “GMCR+” ranks the feasible states according to each decision maker’s preference priority as follows.

- DM 1: $\left(s_{14}{∼}_{1}s_{15}{∼}_{1}s_{16}\right){≻}_{1}\left(s_{5}{∼}_{1}s_{10}\right){≻}_{1}\left(s_{4}{∼}_{1}s_{9}\right){≻}_{1}\left(s_{3}{∼}_{1}s_{8}\right){≻}_{1}s_{13}{≻}_{1}s_{12}{≻}_{1}s_{11}{≻}_{1}\left(s_{2}{∼}_{1}s_{7}\right){≻}_{1}\left(s_{1}{≻}_{1}s_{6}\right)$
- DM 2: $\left(s_{16}{∼}_{2}s_{15}{∼}_{2}s_{14}\right){≻}_{2}s_{10}{≻}_{2}\left(s_{9}{∼}_{2}s_{8}\right){≻}_{2}s_{5}{≻}_{2}\left(s_{4}{∼}_{2}s_{3}\right){≻}_{2}s_{13}{≻}_{2}\left(s_{12}{∼}_{2}s_{11}\right){≻}_{2}s_{7}{≻}_{2}s_{6}{≻}_{2}s_{2}{≻}_{2}s_{1}$
- DM 3: $s_{11}{≻}_{3}s_{12}{≻}_{3}s_{13}{≻}_{3}s_{6}{≻}_{3}s_{1}{≻}_{3}s_{7}{≻}_{3}s_{2}{≻}_{3}s_{14}{≻}_{3}s_{8}{≻}_{3}s_{3}{≻}_{3}s_{15}{≻}_{3}s_{16}{≻}_{3}s_{9}{≻}_{3}s_{4}{≻}_{3}s_{10}{≻}_{3}s_{5}$
- DM 4: $s_{3}{≻}_{4}s_{8}{≻}_{4}s_{4}{≻}_{4}s_{1}{≻}_{4}s_{5}{≻}_{4}s_{2}{≻}_{4}s_{9}{≻}_{4}s_{6}{≻}_{4}s_{10}{≻}_{4}s_{7}{≻}_{4}s_{14}{≻}_{4}s_{11}{≻}_{4}s_{15}{≻}_{4}s_{12}{≻}_{4}s_{16}{≻}_{4}s_{13}$

**Figure 10:**
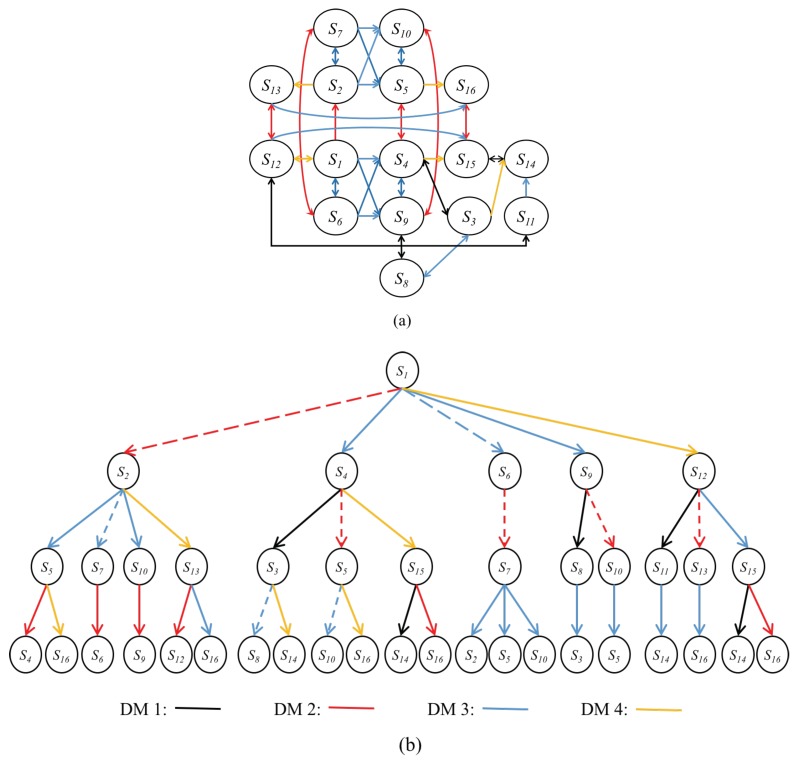
Graph model for the Changzhou conflict. (**a**) corresponding integrated graph; (**b**) three-step tree graph beginning from the status quo (s1).

With the completion of the above steps, the conflict can be modeled in GMCR, and the corresponding integrated graph is shown in Figure 10a, where arrows depict the moves of decision makers among feasible states. Figure 10b is a three-step tree graph beginning from the status quo ($s_{1}$). As can be seen, all other states can be achieved in this tree graph, where the dashed arrows represent unilateral improvements.

**Figure 11:**
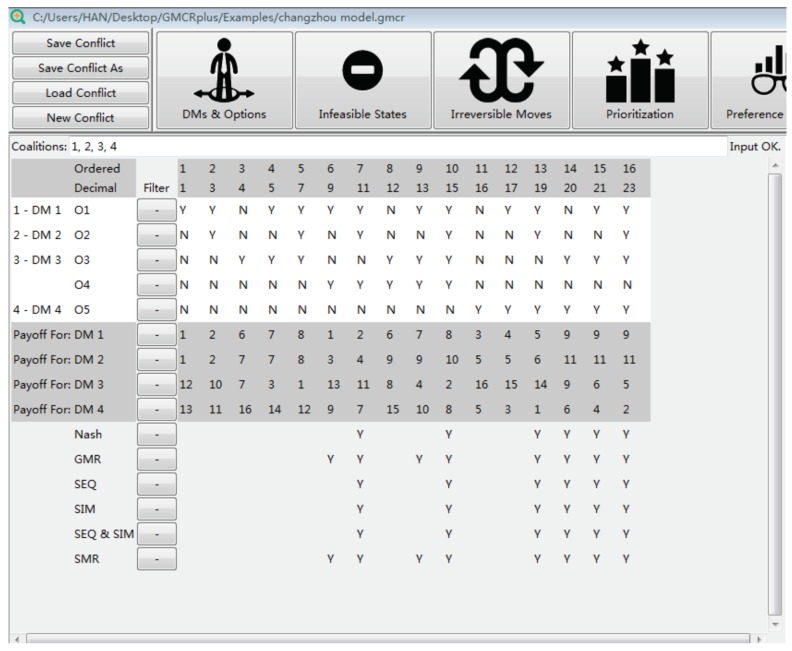
Stability analysis.

Figure 11 lists all possible equilibria. As indicated by the check marks, $s_{7}$, $s_{10}$, $s_{13}$, $s_{14}$, $s_{15}$, and $s_{16}$ are the most stable ones because they satisfy all solution concepts. However, not all equilibria can be achieved in reality. Figure 10b can be used to conduct the status quo analysis to present the evolution path from $s_{1}$ to all equilibria. As shown, only $s_{7}$ can be achieved by unilateral improvements, while other equilibria are unreachable.

Although $s_{7}$ is a strong equilibrium, the conflict continues to persist because DM 1 would never accept the fact that DM 4 does not improve the situation, and DM 3 does not relocate the campus. The nonacceptance of $s_{7}$ forces the conflict to proceed to Stage II, where a third party is needed to intervene in the conflict.

### 4.2. Stage II: Inverse GMCR for Third Party Intervention

In this stage, China’s Ministry of Environmental Protection and Jiangsu Provincial Government cooperated to set up a Joint Investigation Team (JIT) to facilitate the resolution of this incident. JIT has more power than any stakeholder of the conflict. Considering the multiple impacts on society, development, environment, and economic viability, JIT would prefer that the DM 4 can improve the current situation, so that DM 1 and DM 2 take no actions. Both $s_{11}$ and $s_{14}$ satisfy this condition, while $s_{11}$ is much easier to reach because of the complexity of relocating a school. Therefore, $s_{11}$ is the most desired outcome of this case.

**Figure 12:**
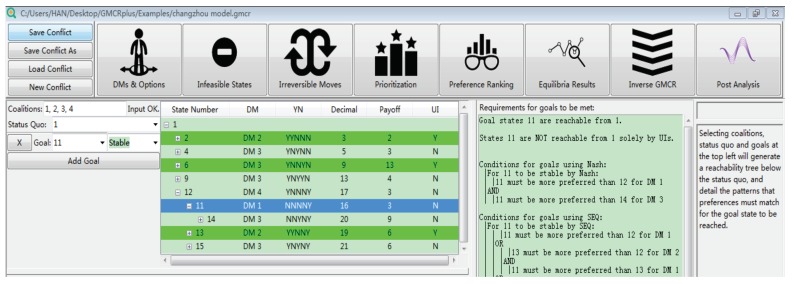
Reachability test.

**Figure 13:**
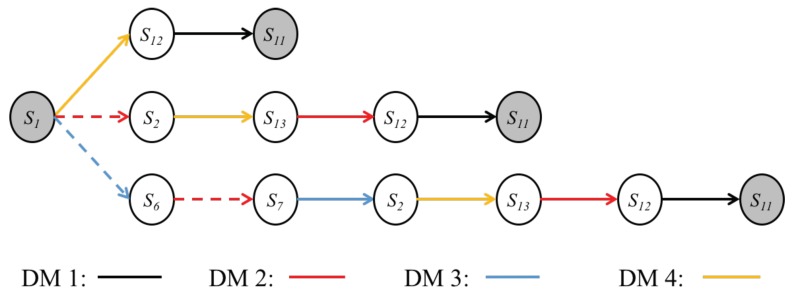
Possible evolution paths.

Thinking from the inverse perspective, to make $s_{11}$ a reachable equilibrium, two conditions must be met: (1) $s_{11}$ satisfies the definition of equilibria; and (2) there is at least one evolution path to reach $s_{11}$ from $s_{1}$. The “Inverse GMCR” on the toolbar of “GMCR+” can help to make the essential preference profiles certain. However, it does not show the evolution path to reach the desired equilibrium. As a supplemental function, the “Post Analysis” can help to show the possible paths from the status quo to a desired state. Figure 12 shows how to use this function. On the left side, the “Status Quo” was set as being $s_{1}$, and then, $s_{11}$ was taken as “Stable”. The third column shows the essential conditions to make $s_{11}$ stable for all decision makers. In this paper, only the Nash stability was considered because it is the most stable one. Two essential conditions to make $s_{11}$ a Nash are shown as follows: (I) $s_{11}$ must be more preferred than $s_{12}$ for DM 1; and (II) $s_{11}$ must be more preferred than $s_{14}$ for DM 3. In the second column, each plus sign represents a move, and the green rows show the unilateral improvement considering the original preference order. Figure 13 shows three possible evolution paths, where dashed arrows represent the original unilateral moves and solid arrows indicate the moves that require preference adjustments.

### 4.3. Discussion of the Intervention Paths

In this section, we discuss in detail how the required and adjustments of preferences can support the third party to facilitate the resolution of this dispute. Referring back to Figure 13, the three evolution paths can provide valuable strategic insights to help the third party (JIT) resolve the dispute among the stakeholders. More specifically, all the solid arrows in this figure need to be treated by adjusting corresponding preferences. The conditions that need to be met for all paths are summarized in Table 6, where the meaning of all conditions is described in the last column.

#### 4.3.1. Condition (A)

For all three paths, Conditions I and II must be satisfied as these two conditions are required for Nash. Condition I indicates that $s_{11}$ should be more preferred than $s_{12}$ for DM 1, i.e., when BPC accepts to improve the current situation, students and their parents prefer to give up their resistance, rather than taking certain measures. It is evident that DM 1 chooses to take measures against BPC because DM 1 does not trust that BPC can really improve the situation as promised. To ensure the environmental problem is solved and hence relieve the distrust from DM 1, the third party may set up a special independent monitoring team, which may include experts and also a certain number of victim representatives. By doing so, DM 1 can be involved in every step of the remediation process, and therefore would not conduct extra activities.

Condition II represents that $s_{11}$ should be more preferable than $s_{14}$ for DM 3, in other words, when BPC decides to improve the current situation, the Changzhou community prefers to abandon the relocation option. This condition is consistent with the original preferences, and so, no extra adjustment need to be done.

#### 4.3.2. Condition (B)

Next, recommendations for satisfying Condition (B) are discussed in terms of each possible evolution path.

##### The Top Evolution Path

If the top evolution path is feasible, Condition III ($s_{12}$ is preferred more than $s_{1}$ for DM 4) has to be met. That means when students and their parents begin to take safeguard measures, BPC prefers to choose the improvement options. The original remediation plan was to prepare the site for a commercial center, which is extremely time-consuming and required complex processes. However, due to the high negative social impact and high health risk, the original remediation plan may not be able to show improvement immediately. Therefore, JIT may communicate with professional organizations, as well as victims and BPC, and suggest a revised remediation plan. For example, changing the plan from ex situ remediation to in situ remediation can substantially cut down the project time and reduce the possibility of secondary pollution because a closed environment is no longer needed. With an agreement among all stakeholders, JIT may be able to persuade DM 4 to adjust its preference and so to satisfy Condition III.

Please note that the above recommendation is only for a short-term resolution. For the long run, adding detailed clauses and increasing the level of compensation and punishment in the environmental legal system is the most effective and necessary way.

##### The Middle Evolution Path

If the middle evolution path is selected, both Condition IV ($s_{13}$ is preferred more than $s_{2}$ for DM 4) and Condition V ($s_{12}$ is preferred more than $s_{13}$ for DM 2) have to be met. In Condition IV, BPC should prefer to take the improvement option when the students and their parents decide to take safeguard measures, and at the same time, CFLS decides to suspend courses. The analysis of this condition follows the previous condition. In Condition V, CFLS prefers to give up the suspending courses option when BPC decides to improve, and at the same time, students and their parents do not trust BPC and continue to take safeguard measures. On the one hand, the main purpose of suspending courses for CFLS is to gain public attention, so that the surrounding environmental and health risks can be reduced. When students and their parents do not trust BPC’s improvement option, CFLS prefers to suspend courses to support the safeguard option. On the other hand, CFLS is a famous private high school because of its high rate of university enrollment. The school does not hope to suspend courses for a long time, which may largely impact the teaching schedule. Considering both aspects, it is possible for JIT to influence CFLS to change its preference. First, an independent monitoring team from different sources is essential to increase the trust, so that the students do not resist attending courses. Second, JIT may guide potential parties (such as CFLS, the parents, the environmental organizations, and even JIT itself) to raise donations for students to purchase water filters and air purifiers. Third, JIT may also provide technical support to CFLS for carrying out a long-distance teaching approach.

##### The Bottom Evolution Path

If the bottom evolution path is feasible, Condition IV, Condition V, and Condition VI ($s_{7}$ is preferred more than $s_{2}$ for DM 3) have to be met. As the first two conditions have been discussed in the previous paragraph, we only consider Condition VI here. In this condition, students and their parents take safeguard measures, CFLS suspends courses, and BPC takes no improvement option, but the Changzhou community does not punish BPC. It is obvious that this situation is impossible to reach, and thereafter, this evolution path is not feasible.

To sum up, it is possible to reach $s_{11}$ from $s_{1}$ through the top and middle evolution path, while the bottom evolution path is unfeasible because Condition VI is hard in practice. It should be noted that the difficulties become greater for JIT, along with the increasing number of preferences that need to be changed.

### 4.4. The Validity of the Proposed Framework

**Figure 14:**
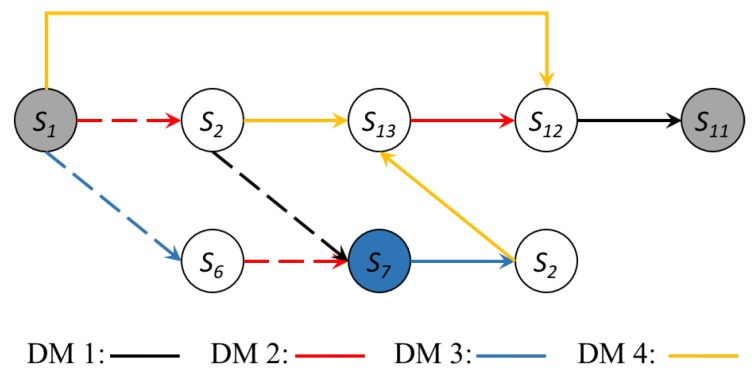
Actual evolution paths.

Compared with the actual transition of the conflict further demonstrates the benefit of using the proposed framework. Figure 14 depicts the actual evolution paths of the Changzhou conflict, where the dashed arrows indicate the moves in Stage I and the solid arrows show those of Stage II.

In more detail, the conflict started in December 2015, when the students were showing symptoms caused by pollution, and their parents took various measures, hoping to solve the problem immediately. This situation was reflected by state $s_{1}$, which is addressed as the status quo. As the situation progressed, the conflict was stable at state $s_{7}$, where the parents were still taking measures, the school suspended courses, and the city municipality chose to punish BPC. Exactly as occurred after the news break-out, this state indicates that the conflict cannot be resolved by the stakeholders themselves through negotiation (Stage I). Particularly, during the five-month period, the conflict continued to escalate, and even reported by CCTV. It has received widespread concerns from the entire country and induced a negative social impact. When the conflict entered Stage II, a third party (JIT) selected state $s_{11}$ as the desired equilibrium, and therefore, the bottom path in Figure 12 gives the evolution path at this stage. The analysis in the previous section provides detailed suggestions to the third party in effectively achieving the proposed outcome.

From the above analysis, the influential role of a third party in BCs is clarified. It is evident that our decision framework lays theoretical foundations for the processes of negotiation and third party intervention, which can be used as an extremely helpful tool to resolve brownfield-related conflicts.

## 5. Conclusions

In recent years, conflicts induced by brownfield incidents have occurred more frequently, especially in developing countries. Brownfield-related conflicts are generally complicated because they may contain economic, health, social, and environmental problems at the same time. A comprehensive method is in urgent need to resolve this type of conflict. Based on the GMCR theory, a two-stage decision making framework was designed to assist both the stakeholders and the potential third parties by providing invaluable strategic insights, and therefore to enhance the understanding and providing potential resolutions for brownfield-related conflicts.

Specifically, the framework was divided into two stages. In Stage I, a forward GMCR model can be built to simulate the moves and counter-moves of stakeholders. By determining the preference relationships of all states for each stakeholder and conducting status quo analysis, the equilibria and evolution paths can be obtained to predict possible resolutions of a brownfield-related conflict. In some cases, the predicted equilibria cannot be accepted by certain stakeholders, and then, the conflict may enter into a deadlock. The longer the conflict continues, the greater the negative impact is. To solve this problem, an inverse GMCR model can be constructed in Stage II for potential third parties to intervene in a brownfield-related conflict. Considering the demands of all stakeholders, a third party should determine one or more states as being desired equilibria. When detecting the requested preference relationships, two aspects should be analyzed: (1) how can a state be an equilibrium? (2) which preferences need to be adjusted on the evolution paths? Based on the obtained information, third parties can take measures to guide the development of a conflict. A recent BC in China, the CFLS toxic incident, was taken as an example to illustrate the effectiveness of the proposed framework. Both forward and inverse analyses were conducted, and several detailed strategies were suggested to facilitate the third party’s intervention process.

To a broader extent, the proposed framework can be applied to brownfield conflicts in other countries or among different countries. Given the flexibility of the graph model for conflict resolution, no modification needs to be conducted to the framework itself. However, the conflict model (such as involved decision makers, options, and preferences) and the format of third-party intervention may need to be adjusted according to various situations.

## Figures and Tables

**Table 1:** The characteristics of stabilities [37,38]. GMR, General Metarationality; SMR, Symmetric Metarationality; SEQ, Sequential Stability.

Stabilities	Preference Information	Foresight	Disimprovement
Nash	Own	Low (1 move)	Never
GMR	All	Medium (2 moves)	Sanction only
SMR	All	Medium (3 moves)	Sanction only
SEQ	All	Medium (2 moves)	Never

**Table 2:** Comparison of forward and inverse GMCR.

Functions	Forward GMCR	Inverse GMCR
Purpose	Predict possible resolutions by determining equilibria	Support a specific resolution to be realized
Required preferences	Full information is required	Minimal information/no information at all
Analysis type	Static stability analysis	Dynamic analysis
Output	Equilibrium results	Scenarios and a set of possible preference patterns
Sensitivity Analysis	Must be performed independently	Inherent

**Table 3:** Decision makers and their options.

Decision Makers	Options	Descriptions
DM 1	$O_{1}$	Take safeguard measures: several available measures may be taken, such as media exposure, protest, petition, and supervision of the remediation process.
DM 2	$O_{2}$	Suspend courses in the new campus: shut down the school or change a safe classroom temporarily until the health risk is eliminated.
DM 3	$O_{3}$	Relocate: relocate the new campus to a new safe site.
	$O_{4}$	Punish: punish BPC if it cannot reduce the health risk to an acceptable range.
DM 4	$O_{5}$	Improve: take measures to improve the current situation.

**Table 4:** Feasible states.

		$s_{1}$	$s_{2}$	$s_{3}$	$s_{4}$	$s_{5}$	$s_{6}$	$s_{7}$	$s_{8}$	$s_{9}$	$s_{10}$	$s_{11}$	$s_{12}$	$s_{13}$	$s_{14}$	$s_{15}$	$s_{16}$
DM 1	1	*Y*	*Y*	*N*	*Y*	*Y*	*Y*	*Y*	*N*	*Y*	*Y*	*N*	*Y*	*Y*	*N*	*Y*	*Y*
DM 2	2	*N*	*Y*	*N*	*N*	*Y*	*N*	*Y*	*N*	*N*	*Y*	*N*	*N*	*Y*	*N*	*N*	*Y*
DM 3	3	*N*	*N*	*Y*	*Y*	*Y*	*N*	*N*	*Y*	*Y*	*Y*	*N*	*N*	*N*	*Y*	*Y*	*Y*
	4	*N*	*N*	*N*	*N*	*N*	*Y*	*Y*	*Y*	*Y*	*Y*	*N*	*N*	*N*	*N*	*N*	*N*
DM 4	5	*N*	*N*	*N*	*N*	*N*	*N*	*N*	*N*	*N*	*N*	*Y*	*Y*	*Y*	*Y*	*Y*	*Y*

**Table 5:** Preferences statements for ranking states.

Decision Makers	DM 1	DM 2	DM3	DM 4
Preference Statements	3	3	−3	−5
	5	5	−1	−1
	1IF(−3)	4	5	−4
	1IF(−5)	2IF(−3)	−2	−2
	2IF(−3)	2IF(−5)	4	3
	2IF(−5)			

**Table 6:** Preference statements for ranking states.

Evolution Paths	Condition (A)	Condition (B)	Descriptions
The Top Evolution Path	I, II	III	I: $s_{11}{≻}_{1}s_{12}$, II: $s_{11}{≻}_{3}s_{14}$, III: $s_{12}{≻}_{4}s_{1}$, IV: $s_{13}{≻}_{4}s_{2}$, V: $s_{12}{≻}_{2}s_{13}$, VI: $s_{7}{≻}_{3}s_{2}$
The Middle Evolution Path		IV, V	
The Bottom Evolution Path		IV, V, VI	
